# 

**Published:** 1865-09

**Authors:** 


Volume XXII. Number 9.

### THE CHICAGO MEDICAL JOURNAL

De LASKIE MILLER, M. D.,
EFHRAIM INGALS, M. D.,
Editors and Proprietors.

SEPTEMBER, 1865.

CHICAGO:

J. C. W. BAILEY, BOOK AND JOB PRINTER, 164 CLARK STREET.

1865.

